# Eosinophilic Granulomatosis With Polyangiitis Presentation After Nandrolone Withdrawal

**DOI:** 10.7759/cureus.42973

**Published:** 2023-08-04

**Authors:** Maria J Garcia-Blanco, Francisco-Javier Rodeles, Laura Muñoz, Sergio Lopez-Anguita, Raul Ruiz-Esteban

**Affiliations:** 1 Department of Internal Medicine, Hospital Central de la Defensa - Gomez Ulla, Madrid, ESP; 2 Medicine, Universidad de Alcala, Alcalá de Henares, ESP; 3 Department of Neurology, Hospital Central de la Defensa - Gomez Ulla, Madrid, ESP

**Keywords:** anca negative vasculitis, anca associated vasculitis, autoimmune vasculitis, nandrolone, eosinophilic granulomatosis with polyangiitis (egpa)

## Abstract

A 41-year-old man was admitted to the Emergency Department with bilateral numbness in lower extremities and left femoral palsy. He also reported constitutional symptoms, vomiting and non-bloody diarrhoea for the last several months. He had a previous history of eosinophilic asthma with erratic follow-up. During admission, eosinophil count was 66% of white blood cells. Sural nerve biopsy revealed vasculitis with eosinophilic infiltration. Further evaluations consisted of colonoscopy and nasal endoscopy that confirmed eosinophil infiltrates on colonic ulcers and nasal polypi. The patient was started on systemic corticosteroids and cyclophosphamide. Among his personal records, he had been taking nandrolone decanoate without medical prescription, and had withdrawn a few years before the first asthma exacerbation.

## Introduction

Eosinophilic granulomatosis with polyangiitis (EGPA; formerly called Churg-Strauss syndrome) is a vasculitis targeting the small and medium-sized arteries and can affect multiple organ systems [1]. EGPA typically appears in three sequential clinical phases. First, the prodromal phase whose main symptoms are rhinitis, atopy and asthma. The eosinophilic phase consists of peripheral blood eosinophilia and organ eosinophilic infiltration, especially lung and gastrointestinal tract. And last, the vasculitic phase usually presents with constitutional symptoms [2].

Among other vasculitides, EGPA is relatively uncommon [3] and its prevalence in Western countries ranges from 10.7 to 22.3 cases/million [4]. The pathogenesis of EGPA is unknown but is characterized by a disbalance in immune function [5]. EGPA has been associated with several medications for the treatment of asthma, such as leukotriene modifying agents, inhaled glucocorticoids and omalizumab. Nevertheless, it remains unclear whether this relationship is pathogenic or if prodromal symptoms of EGPA appear due to lowering the dose of systemic glucocorticoids when they are introduced [6].

Anabolic steroids are part of the treatment for several medical conditions, such as osteoporosis or malnutrition. However, non-medical use of anabolic steroids is commonplace among bodybuilders and powerlifters. For instance, nandrolone is an androgen and anabolic steroid but also has effects on immune system activation [7].

## Case presentation

A 41-year-old male patient presented to the emergency department with progressive numbness involving both lower extremities and weakness and pain in the left lower extremity. He also reported weight loss, fatigue, weakness, loss of appetite, nausea and vomiting, and non-bloody diarrhoea for the last several months. He otherwise denied dyspnoea, fever, skin lesions, pruritus and saddle anaesthesia.

On examination, the patient was febrile to 38ºC, had 3+/5 strength in the left ankle extensors and 1+/5 in flexors, left ankle oedema, anaesthesia of left foot and lateral aspect of the right foot. Both left rotulian and Achilles reflex were abolished.

**Figure 1:**
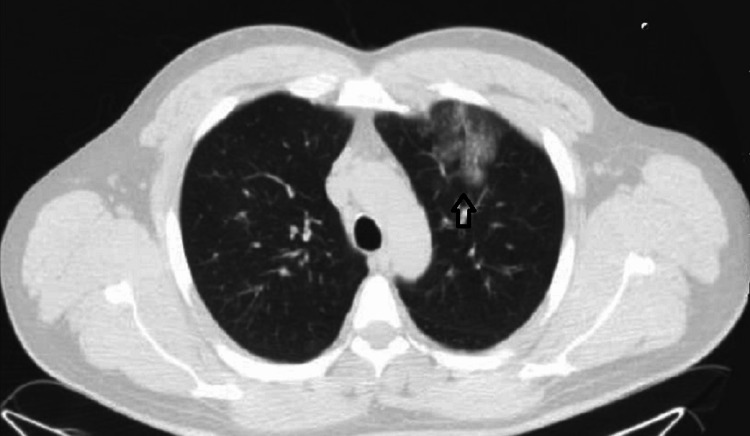
Chest computed tomography scan on previous admission Chest computed tomography scan showing pulmonary infiltrate during a previous admission because of severe asthma exacerbation.

The patient had a personal history of eosinophylic asthma with three exacerbations that had required hospital admission in the last two years. In all these opportunities the patient was commenced on intravenous methylprednisolone with good clinical response and resolution of eosinophilia, being discharged with oral prednisone tapering and inhaled combination of long-acting beta-agonists (LABA) and inhaled corticosteroids (ICS). However, he had always discontinued treatment and lost follow-up. In the last admission six months before presentation, a computed tomography (CT) scan revealed pulmonary opacities (Figure 1). And transbronchial pulmonary biopsy and bronchial aspirate showed unspecific chronic inflammation. Both IgG and IgE for Aspergillus fumigatus levels were normal. 

The patient was raised in rural Central America and had also lived in the United States where he had performed as a competitive bodybuilder. He finally moved to Spain a few years before presentation and worked as a bricklayer. As a child, he had had a helminth infection that was treated with an antiparasitic agent whose name he did not recall. When asked for any other medications in the past, he reported that he had been taking nandrolone decanoate for years for improving his physique without medical control. Withdrawal occurred when he moved to Spain, a few years before the first asthma exacerbation. He denied having taken any other drug, prescription medicine or nutritional supplement.

At the emergency department, blood test showed significant leukocytosis with eosinophilia. Renal function and liver tests were normal. Chest x-ray revealed no pulmonary consolidations nor pleural effusions and lower extremities ultrasonography ruled out deep venous thrombi. With diagnosis of hypereosinophilic syndrome, the patient was transferred to the Internal Medicine unit to pursue further investigations.

The primary differential diagnoses at that point were EGPA and helminthic infection. Eosinophilic leukemia was also considered.

Autoimmune screening tested negative, as shown in Table 1, along with microbiological test results. Blood smear showed prominence of eosinophilia with no atypical lymphoid cells. Both hypothalamic-pituitary-adrenal and thyroid axes function tested normal. On visual examination, bone marrow was markedly hypercellular with abundant eosinophils in all stages of maturation, without dysplastic eosinophils nor blasts, and without neoplastic nor helminthic infiltration. JAK-2 and BCR/ABL mutations tested negative.

**Table 1:** Laboratory test results PCR: Polymerase Chain Reaction

Microbiology tests	Test results	Reference value
Epstein-Barr Virus		
IgM	Negative	
IgG	Positive	
Hepatitis A Virus		
IgM	Negative	
IgG	Positive	
Hepatitis B Virus (HBV)		<0.5 UI/L
HBsAg	Negative	
Anti-HBs	1.51 UI/L	
Anti-core	Negative	
Anti-Hepatitis C Virus (HCV) Antibodies	Negative	
Human Immunodeficiency Virus (HIV)		
Anti-HIV	Negative	
p24 antigen	Negative	
Syphilis Treponemic test	Negative	
Toxoplasma gondii		
IgM	Negative	<10 UI/mL
IgG	40.60 UI/mL	
Strongyloides stercolaris	Negative	
Quantiferon	Negative	
Blood culture	Negative	
Induced sputum culture	Negative	
Cerebrospinal fluid culture	Negative	
Stool samples		
Helminthic visualisation (x3)	Negative	
Stool culture for bacteria	Negative	
Strongyloides stercolaris PCR	Negative	
Protozoal PCR	Negative	
Helicobacter pylori antigen stool test	Negative	
Immunology		
Anti-PR3 Antibodies	Negative	
Anti-Myeloperoxidase (MPO) Antibodies	Negative	
Antinuclear Antibodies (ANA)	Negative	
Anti-Glomerular Basal Membrane antibodies	Negative	
Cryoglobulin	Negative	
Rheumatoid factor	794 U/mL	0.1-14 U/mL
IgE	4236.00 kU/L	33-188 kU/L
Tryptase	11.5 ug/L	0-18 ug/L

CT of the chest revealed mild bronchial wall thickening but no pulmonary opacities. Transthoracic echocardiography revealed no restriction nor any other data of eosinophil infiltration of myocardium. Nasal endoscopy revealed nasal polyposis, and colonoscopy showed colonic ulcers. Indeed, eosinophil infiltration was found on biopsy of both colonic ulcers and nasal polyposis.

Within 24 hours of admission, magnetic resonance of the central nervous system ruled out spinal cord compression. No eosinophils were detected in cerebrospinal fluid. Nerve conduction study and electromyography revealed mononeuritis multiplex with axonal, distal and asymmetrical sensorial damage in both lower limbs, and motor weakness of the left common peroneal nerve.

The biopsy of the left sural nerve revealed acute vasculitic activity with eosinophilic infiltrate. The architecture of nerve fascicles was conserved. There was prominent perivascular inflammatory infiltrate with predominance of lymphocytes and eosinophils in the epineurium. An epineural vessel was affected with transmural inflammatory infiltrate with karyorrhexis and fibrinoid necrosis, along with destruction of the vascular wall with disruption of the endothelium, elastic laminae and smooth muscle. Vessels showed no sign of obliteration nor recanalization. No granulomas or amyloid deposit were observed. Acute axonal degeneration was present in all the studied nerve fascicles, with severe endoneural macrophage infiltration. There was also a relevant loss of both myelinated and amyelinated fibers, as well as acute degeneration of myelinated fibers.

Clinically, symptoms of hypereosinophilia are caused by eosinophilic tissue infiltration and the organs affected regardless of the etiology of eosinophilia. The initial management of eosinophilia of unknown etiology includes empiric deworming, especially in patients coming from areas where nematodes are endemic as in this case [8]. Therefore, the patient received ivermectin 400 mg/kg and albendazole 400 mg single dose [8]. Immediately afterwards, he was commenced on systemic glucocorticoids, first methylprednisolone 500 mg once daily for three days, followed by prednisone taper starting at 60 mg per day. Eosinophil count evolution is shown in Tables 2, 3.

**Table 2:** Eosinophil count before presentation bp: before presentation, MTP: methylprednisolone

	Asthma exacerbation		Asthma exacerbation		Asthma exacerbation	
	23 months bp		18 months bp		8 months bp	
Eosinophils (normal range)	Day 0	Day +4	Day 0	Day +1	Day 0	Day +1
	MTP 80 mg		MTP 60 mg		MTP 60 mg	
% (0-7)	18.8	0.1	9.5	0.1	11.4	0.9
Count (10³/µl) (0-0.7)	1.24	0.01	1.11	0.01	0.94	0.11

**Table 3:** Timeline of eosinophil count ap: after presentation, CPM: cyclophosphamide, MTP: methylprednisolone, MTX: methotrexate, PDN: prednisone

	Presentation						Discharge		
Eosinophils (normal range)	Day 0	Day +4	Day +8	Day +14	Day +26	Day +31	Day +36	3 months ap	6 months ap
		MTP 500 mg (days 3-5)	PDN 30 mg (from day +6)	PDN 15 mg	CPM 500 mg + PDN 15 mg			MTX 15 mg weekly + PDN 5 mg	
% (0-7)	66.6	9.6	13	1.5	15.5	0	0.1	12.6	7.5
Count (10³/µl) (0-0.7)	27.75	2.32	1.73	0.17	1.41	0	0.02	0.81	0.64

The American College of Rheumatology (ACR) defined six criteria for the classification of EGPA if vasculitis is histologically documented [9]: asthma, white blood cell count with eosinophil count >10%, mononeuritis multiplex, transient pulmonary opacities, paranasal sinus abnormality and vasculitis with eosinophil infiltrate on sural nerve. This patient met all six criteria regarding the results of the biopsy of the sural nerve.

Given the neurologic involvement, an immunomodulatory agent was added to glucocorticoids for remission induction. The agent chosen was cyclophosphamide because of its widespread use and extensive experience in multiorgan system affection, especially in antineutrophil cytoplasmic antibody (ANCA)-negative patients such as our patient [10]. The patient was also recommenced on budesonide and formoterol before discharge. An ambulatory rehabilitation and physiotherapy plan was started for improving impaired mobility and quality of life, with an ankle orthosis and physical exercise.

After discharge, the patient continued follow-up at the autoimmune diseases unit at the internal medicine department. After eight sequential doses every two weeks of cyclophosphamide, remission was achieved, and patient was switched to subcutaneous methotrexate and a daily low dose of oral prednisone. He also does follow-up at the respiratory department and is on inhaled ICS/LABA.

Electromyography four months after presentation has not shown great improvement. Whereas femoral nerve palsy persists, the patient can walk without orthosis. Although he has changed jobs, he has returned to work and to the gym.

There has been no relapse of EGPA, eosinophil count persists within normal range (Table 2) and eosinophilic asthma is well controlled without exacerbation during these months.

## Discussion

EGPA is a multisystem vasculitis whose mechanism is not well known, although abnormal immune function is key to its development and presentation of the symptoms. T-regulatory cells increase IL-10 production when EGPA relapses and decrease when remission is achieved [11]. Substantial environmental factors have been related to EGPA, especially air pollutants and silicon dioxide [12]. Also, some drugs such as cocaine, levamisole and methimazole have been associated with EGPA, mainly through a mechanism of hypersensitivity and IgE activation [12]. Our patient denied having taken any of these, as written above.

Nandrolone is an androgenic anabolic steroid with several medical indications such as malnutrition. Researchers are developing nandrolone-esters that reduce its secondary effects because of how nandrolone affects the sexual-hormone axis [13]. On the other hand, nandrolone is widely used among bodybuilders since it helps with muscle gain, as was the case of our patient.

Besides its anabolic effects, nandrolone modifies the immune response although its role is not clearly established yet. Nandrolone has been reported to produce immunity dysregulation by enhancing immune response, cytotoxicity and inflammation in mice [14]. However, according to other published works, nandrolone administration may act as a healing inhibitor [15] and seems to suppress inflammatory cytokine gene expression in older rats during functional overload [16]. Whether nandrolone may have an effect on autoimmune diseases remains unknown although it has been associated with lower autoantibodies synthesis in mice [17]. The modulation of the immune response has also been described in vitro for human lymphocytes [18].

In the case here reported, we theorize about the possibility that the activity of EGPA was diminished while the patient was on nandrolone because its intake would mitigate eosinophil activation. This is a mere hypothesis, but is supported by the fact that the patient presented the first asthma exacerbation that required hospital admission a couple of years after nandrolone withdrawal, which could have been the trigger to immune system dysbalance. As far as we are concerned, there are no studies that establish a clear relationship between nandrolone and immune system dysbalance in humans. Nonetheless and regarding humans, nandrolone non-medical use has already been associated with tuberculosis reactivation [19] and impaired immune system response to tuberculosis, and high eosinophil count [20] in at least two patients. Although the first severe asthma exacerbation of our patient occurred years after nandrolone withdrawal, EGPA symptoms can present clinically years after the onset of altered immune function.

## Conclusions

This is, to the extent of our knowledge, the first case reporting a patient who presented with symptoms of EGPA years after nandrolone withdrawal. No causal relationship can be established to date between this drug and EGPA. However, health providers must be aware of the possible secondary effects regarding the immune system function in patients on anabolic steroids both for medical and non-medical since these drugs they may act as immunosuppressants and obscure symptoms of autoimmune diseases. This could be troublesome if the diagnosis of an autoimmune disease is belated, and the prescription of the accurate treatment is delayed. Further studies should be made in this direction if more cases are reported in the future.
